# The longitudinal cerebrospinal fluid metabolomic profile of amyotrophic lateral sclerosis

**DOI:** 10.3109/21678421.2015.1053490

**Published:** 2015-06-29

**Authors:** Elizabeth Gray, James R. Larkin, Tim D. W. Claridge, Kevin Talbot, Nicola R. Sibson, Martin R. Turner

**Affiliations:** ^a^ Nuffield Department of Clinical Neuroscience, University of Oxford, Oxford, UK; ^b^ Cancer Research UK and Medical Research Council, Oxford Institute for Radiation Oncology, Department of Oncology, University of Oxford, Oxford, UK; ^c^ Department of Chemistry, University of Oxford, Oxford, UK

**Keywords:** Nuclear magnetic resonance, proton, motor neuron disease, neurochemical, biomarker

## Abstract

Neurochemical biomarkers are urgently sought in ALS. Metabolomic analysis of cerebrospinal fluid (CSF) using proton nuclear magnetic resonance (^1^H-NMR) spectroscopy is a highly sensitive method capable of revealing nervous system cellular pathology. The ^1^H-NMR CSF metabolomic signature of ALS was sought in a longitudinal cohort. Six-monthly serial collection was performed in ALS patients across a range of clinical sub-types (*n = *41) for up to two years, and in healthy controls at a single time-point (*n = *14). A multivariate statistical approach, partial least squares discriminant analysis, was used to determine differences between the NMR spectra from patients and controls. Significantly predictive models were found using those patients with at least one year's interval between recruitment and the second sample. Glucose, lactate, citric acid and, unexpectedly, ethanol were the discriminating metabolites elevated in ALS. It is concluded that ^1^H-NMR captured the CSF metabolomic signature associated with derangements in cellular energy utilization connected with ALS, and was most prominent in comparisons using patients with longer disease duration. The specific metabolites identified support the concept of a hypercatabolic state, possibly involving mitochondrial dysfunction specifically. Endogenous ethanol in the CSF may be an unrecognized novel marker of neuronal tissue injury in ALS.

## Introduction

Amyotrophic lateral sclerosis (ALS) is a heterogeneous, progressive and uniformly fatal neurodegenerative disease characterized by clinically variable loss of upper and lower motor neurons and associated frontotemporal cerebral pathways. Over the past decade, a number of candidate neurochemical biomarkers have emerged from the analysis of serum and cerebrospinal fluid (CSF) (1,2). Nevertheless, there remains a need for biomarkers that are sensitive to diagnosis in those with atypical clinical features, capable of prognostic stratification and measuring therapeutic response in ALS, as well as in understanding the pathophysiology of the condition.

Characterization of the metabolome offers the potential to develop a disease-specific signature that facilitates sub-group stratification, as well as providing potentially novel insights into deranged biochemical pathways at close proximity to neuropathological tissue. High-throughput metabolomic techniques have been applied to ALS, including high resolution proton nuclear magnetic resonance (^1^H-NMR) spectroscopy (3–5), high performance liquid chromatography (HPLC) followed by electrochemical detection (6) and either gas or liquid chromatography coupled to mass spectrometry (GC-MS, LC-HRMS) (7–10). Significant differences in metabolites have been observed in studies comparing serum and CSF from ALS patients with healthy controls. These have been consistent with a range of established pathogenic mechanisms in ALS, including mitochondrial dysfunction, oxidative stress, excitotoxicity and hypermetabolism (11), but studies have largely been conducted in cross-sectional rather than longitudinal cohorts.

Proton spectroscopy metabolomics has the advantages of limited sample preparation and of being non-destructive. This study applied ^1^H-NMR spectroscopy to identify a signature of CSF metabolites within a longitudinal cohort of apparently sporadic ALS patients. In addition to the potential for biomarker development, determining the identity of the most discriminant molecules compared with healthy controls and between fast and slow progressing patients, offers potential for the identification of novel pathogenic mechanisms.

## Methods

### Participants

Participants were recruited as part of The Oxford Study for Biomarkers in Motor Neuron Disease (BioMOx). CSF samples were obtained serially every six months from prevalent and incident cases of ALS attending The Oxford Motor Neuron Disease Centre, and diagnosed by two experienced neurologists (MRT, KT) according to standard criteria (12). All participants in this analysis were apparently sporadic ALS patients (i.e. not reporting a family history of ALS or frontotemporal dementia). Patients were excluded if they suffered from any other significant medical disorder such as diabetes. Healthy volunteers without signiﬁcant past medical history (spouses and friends of patients) were recruited for a single time-point sampling only. All participants were capable of providing informed consent. The study was approved by the South Central Oxford Ethics Committee B.

Patients were examined at each visit (MRT). Disease duration was calculated in months from symptom onset to date of sampling. Disability was assessed using the revised ALS Functional Rating Score (ALSFRS-R, 0–48, lower score reflects greater disability). Progression rate was calculated in ALSFRS-R score decrease per month as: (48 minus ALSFRS-R)/(disease duration).

### Sample preparation

CSF samples were processed within 1 h of extraction; no CSF samples were contaminated by frank blood and potential debris was removed by centrifugation (4°C, 3000 *g*, 10 min). Samples were stored in polypropylene tubes at –80°C until analysis. Prior to NMR, samples were defrosted at 4°C. Aliquots (100 μl) were diluted in a 5-mm NMR tube to a final volume of 600 μl with 0.24 M sodium phosphate buffer (pH 7.4, 0.1% NaN_3_, 0.8% NaCl) in D_2_O containing 1 mM TSP (3-trimethylsilyl-1-[2,2,3,3,-^2^H_4_] propionate) as an internal standard.

### NMR spectroscopy

Proton NMR spectroscopy was performed as previously described (13). A 700-MHz NMR system (Bruker Avance III equipped with a ^1^H TCI cryoprobe) was used to acquire ^1^H NMR spectra from each sample. A 1D NOESY pre-saturation sequence, with solvent pre-saturation during the relaxation delay (2 s) and mixing time (10 ms) was used for all samples. Automatic baseline correction was performed on all 1D spectra using a 3rd-order polynomial (Topspin 3.2) and all spectra were manually corrected for phase distortion. To assist with metabolite identification, two-dimensional Correlation Spectroscopy (COSY) ^1^H NMR spectra were acquired from one sample within each group. Acquisition of COSY spectra was performed with 1.5-s solvent pre-saturation, a spectral width of 10 ppm (7002 Hz), and 16 or 32 transients per t1 increment for 256 increments. All NMR spectra were acquired at 293 K.

### Data analysis

Prior to data pre-processing, non-linear peak alignment (14) was performed on each 1D ^1^H spectrum. Subsequently, spectra were sub-divided into 0.02-ppm regions (δ = midpoint of integral region) and integrated between 0.2 and 9.6 ppm using a custom MATLAB (MathWorks, Inc.) script. This reduced the spectra to ∼435 independent variables. The region between 4.30 and 5.00 ppm was highly variable due to imperfect water suppression and was excluded to minimize unwanted deterioration of spectral quality. Initially, a singlet at 3.36 ppm was identified as a significant contributor to a number of models. The identity of this peak was confirmed using spiking and HSQC (heteronuclear single quantum coherence) spectroscopy as methanol, a common laboratory contaminant from cleaning glassware. Consequently, the spectral region from 3.34 to 3.38 ppm was excluded from all analyses. PLS-DA was applied to the data following scaling using the Pareto variance to suppress noise.

### Study design

**Figure 1.**
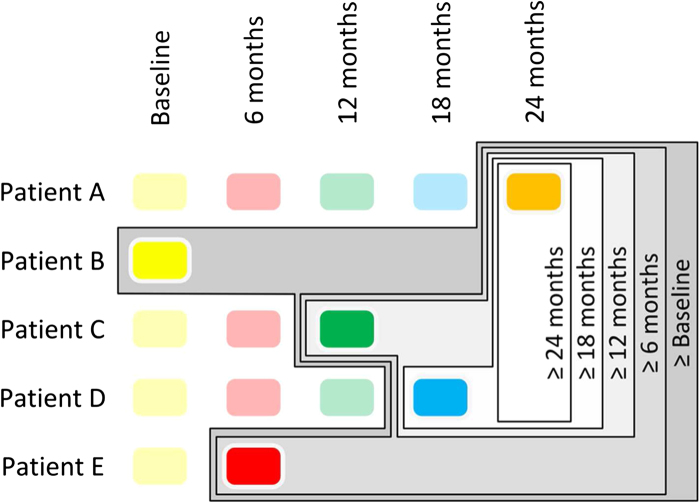
Schematic demonstrating the experimental design.

Five models were built from the study CSF cohort (Figure 1). In each case only the most advanced sample, with respect to disease course, from each patient was used. The ‘≥ 24 months’ model included ALS patients whose most advanced longitudinal sample was ≥ 24 months from study enrolment. The ‘≥ 18 months’ model also included patients whose most advanced sample was ≥ 18 months from study enrolment as did the ‘≥ 12 months’, ‘≥ 6 months’ and ‘≥ Baseline’ models, which contained progressively greater sample numbers owing to follow-up attrition. By constructing the groups in this fashion it was possible to eliminate bias arising from inclusion of multiple samples from the same patient, while maximizing separation from the control cohort. A PLS-DA model was also built separating slow- and fast-progressing ALS patients according to a progression rate below or above 1 point decrease per month, respectively.

### Statistical methods

For each comparison, a partial least squares discriminant analysis (PLS-DA) model was built to best explain differences between the variables for the groups being studied (SIMCA 13.0, Umetrics, Sweden). The *q* ^2^-value was calculated to determine the potential predictive nature of the models. The metric *q* ^2^ is derived from a stepwise cross-validation of the model, whereby a model generated by withholding one-seventh of the samples in seven successive simulations is used to predict group membership of the missing samples. A *q* ^2 > ^0 means the model is predictive, but *q* ^2 > ^0.4 is generally regarded as the threshold for significance in biological modelling (13).

Further validation was carried out using a pseudo-Monte Carlo method where 200 models were built using random group assignments. Models were considered significant where the genuine *q* ^2^ was higher than 95% of the randomly generated *q* ^2^-values. Buckets with a VIP (Variable Importance Plot) score greater than 2 were considered to be the most important for model separation. To identify the metabolites contributing to the spectra in each bucket the relevant resonances were identified using a combination of literature values, COSY spectroscopy, spiking, HSQC spectroscopy and reference to the human CSF metabolome database (15,16).

To assess differences in gender between ALS patients and control volunteers, Fischer's exact tests were performed. A one-way ANOVA with the appropriate post hoc test or Student's *t*-test was used to assess differences in age, CSF protein and glucose concentration between ALS patients and control volunteers.

## Results

**Figure 2.**
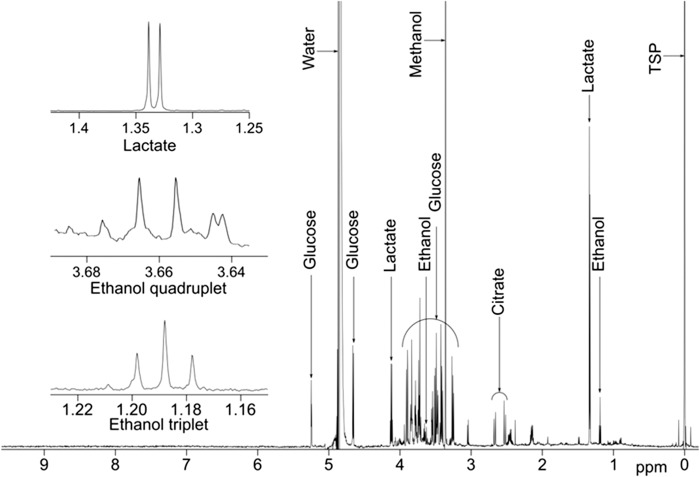
Example ^1^H NMR spectrum of CSF from an ALS patient with key metabolites identified.

An example CSF spectrum with peaks of interest annotated is shown in Figure 2.

### Participants and samples

In total, CSF samples were obtained from 41 ALS patients, five patients with the very slowly-progressive variant of primary lateral sclerosis (PLS) and 14 healthy controls (Table I). No overall group difference in the glucose or protein levels in CSF was found between groups. For each model, no significant differences were present with respect to the gender of ALS patients compared to control volunteers. A significant difference in age was present in the model comparing control samples with all baseline ALS patients.

**Table I.** Characteristics of patients with ALS, PLS and controls.

ALS patient groups							
	≥ Baseline (*n = * 40)	≥ 6 months (*n = * 20)	≥ 12 months (*n = * 14)	≥ 18 months (*n = * 11)	≥ 24 months (*n = * 6)	PLS (*n = * 5)	Controls (*n = * 14)
Gender, *n* (%)							
Female	12 (30%)	6 (30%)	4 (28.6%)	3 (27.3%)	2 (33.3%)	4 (80%)	8 (57.1%)
Male	28 (70%)	14 (70%)	10 (71.4%)	8 (72.7%)	4 (66.7%)	1 (20%)	6 (42.9%)
Age (y)	62.2 ± 10.4*	60.9 ± 11.0	58.8 ± 10.8	56.1 ± 9.3	55.7 ± 11.0	70.2 ± 6.7	53.2 ± 8.9
Disease duration (months)	37.8 ± 32.7	53.2 ± 35.0	66.7 ± 33.3	71.7 ± 35.9	85.4 ± 38.5	162.5 ± 79.4	N/A
ALSFRS-R	30.7 ± 8.3	25.3 ± 7.0	25.3 ± 4.8	25.9 ± 4.9	25.5 ± 4.9	25.8 ± 6.2	N/A
Progression rate	0.9 ± 1.0	0.7 ± 0.6	0.4 ± 0.2	0.4 ± 0.2	0.3 ± 0.2	0.2 ± 0.07	N/A
CSF							
Glucose (mM)	3.9	3.9	4.1	4.2	3.8	3.7	3.7
Protein (mg/ml)	0.97	1.0	1.0	1.0	1.0	0.98	0.95

*p < 0.05 ≥ Baseline compared to controls. Values are mean ± SD.

### CSF spectra multivariate statistical analysis

**Figure 3.**
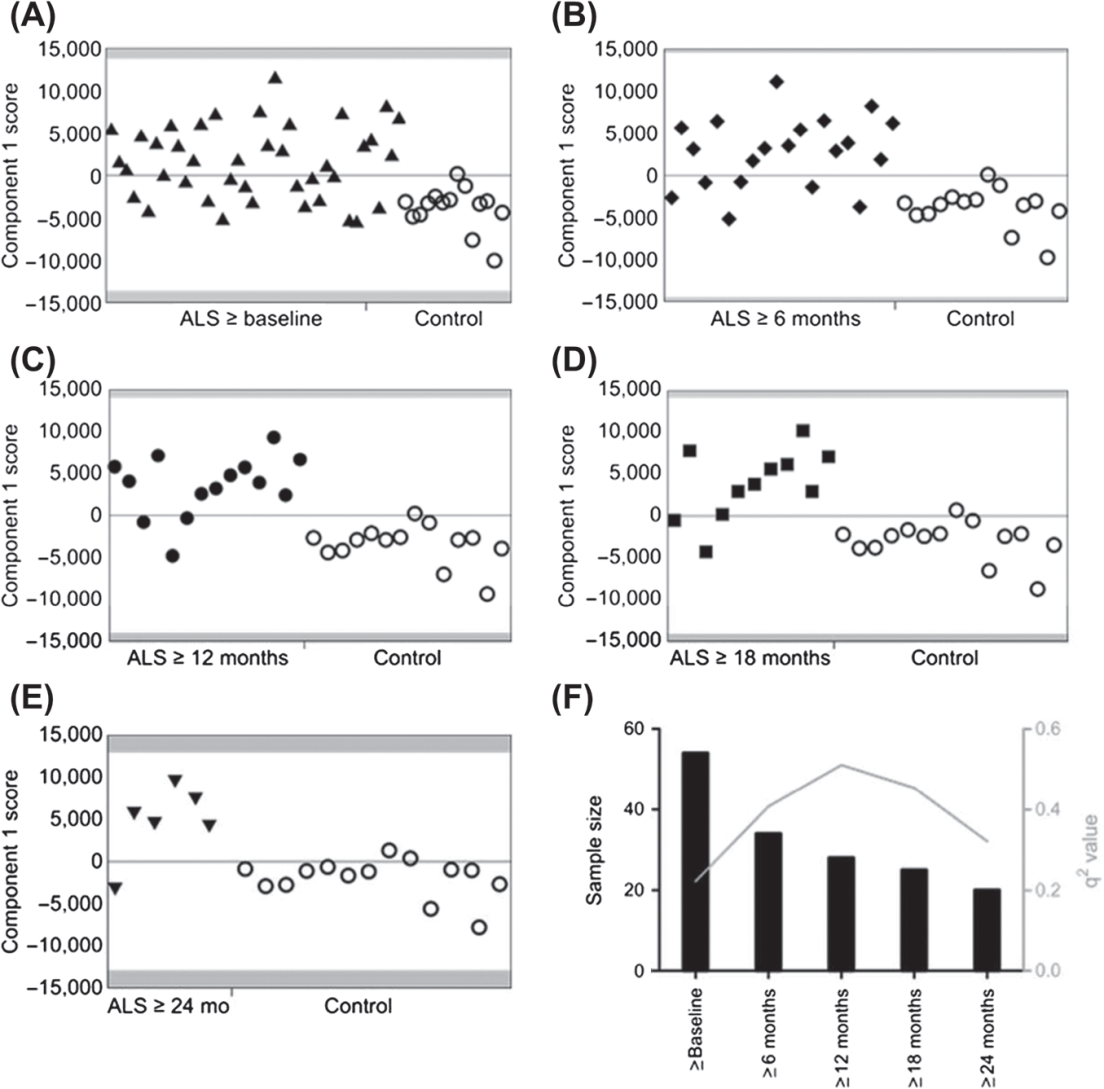
PLS-DA model plots. In each plot, the x-axis indicates patient number while the y-axis indicates discrimination for the model's component. (A) PLS-DA plot of CSF samples comparing ALS patients for whom the most advanced sample is greater than or equal to baseline (filled black triangles) and control volunteers (open black circles). (B) PLS-DA plot of CSF samples comparing ALS patients for whom the most advanced sample is greater than or equal to six months (filled black diamonds) and control volunteers (open black circles). (C) PLS-DA plot of CSF samples comparing ALS patients for whom the most advanced sample is greater than or equal to twelve months (filled black circles) and control volunteers (open black circles). (D) PLS-DA plot of CSF samples comparing ALS patients for whom the most advanced sample is greater than or equal to 18 months (filled black squares) and control volunteers (open black circles). (E) PLS-DA plot of CSF samples comparing ALS patients for whom the most advanced sample is greater than or equal to 24 (inverted filled black triangles) and control volunteers (open black circles). (F) Graph to show the *q* ^2^-values of the models and their corresponding sample size.

Initially, PLS-DA models were constructed separating control volunteers from each of the patient groups, excluding the slowly-progressive PLS patients (Figure 3A–E). The most significantly predictive model was that comparing control samples with the ≥ 12 months subset of ALS patients (*q* ^2^ = 0.51; Figure 3C). The score plot of this one-component PLS-DA model showed good discrimination between the two populations. Examination of the loadings revealed that glucose (a large number of buckets in the range δ_x-y_ = 3.25–3.91), lactate (δ_x-y_ = 1.33 and 4.11–4.13), citric acid (δ_x-y_ = 2.64–2.66), and ethanol (δ_x-y_ = 3.65–3.67 and 1.19) were all increased in ALS patients compared to controls.

Models built separating control volunteers from ALS patient samples taken ≥ 6 months and ≥ 18 months after baseline were also significantly predictive (*q* ^2^ = 0.41 and 0.45, respectively; Figure 3B,D). In each case, examination of the loadings revealed that glucose (a number of buckets in the range δ_x-y_ = 3.25–3.91 ppm), lactate (δ_x-y_ = 1.33 and 4.11–4.13), citric acid (δ_x-y_ = 2.64–2.66), and ethanol (δ_x-y_ = 3.65–3.67 and 1.19), were again increased in ALS patients compared to controls.

A further model separating control volunteers from ALS patient samples taken > 5 years after disease onset was predictive (*q* ^2^ = 0.47). Examination of the loadings revealed that the same metabolites were increased in ALS patients with respect to control volunteers, with the exception of citric acid. Although this latter metabolite was still altered relative to control, its contribution to the positive model was considerably lower than previously.

Two other models, comparing control volunteers to ALS patient samples collected either at baseline or ≥ 24 months from baseline, although positive, failed to achieve significance (*q* ^2^ = 0.22 and 0.32, respectively; Figure 3A,E). Incorporation of samples from the five slowly-progressive PLS patients to each model, however, resulted in a significant separation between control volunteers and the ≥ 24 months ALS patient subset (*q* ^2^ = 0.42). All predictive models were successfully validated using the cross-validation embedded in a Monte-Carlo re-sampling approach (Supplementary Figure 1 to be found online at http://informahealthcare.com/doi/abs/10.3109/21678421.2015.1053490). Figure 3F summarizes *n* and *q* ^2_^ values for each model. A model separating all ALS patients divided into slow (0–1 ASLFRS-R decrease/month) and fast (> 1 ALSFRS-R decrease/month) progressing groups was not predictive, with a *q* ^2^-value of –0.16 (Supplementary Figure 2 to be found online at http://informahealthcare.com/doi/abs/10.3109/21678421.2015.1053490).

### Selected metabolite analysis

**Figure 4.**
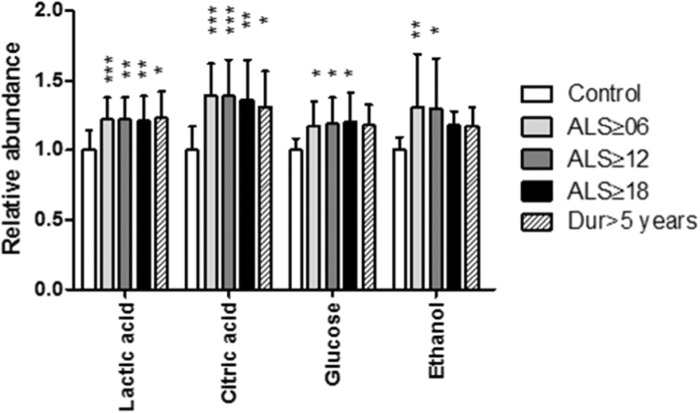
Relative abundance of metabolites, normalized to control and determined by summed integral from the regions indicated in Table II. Data are means ± SD. * = *p < *0.05; ** = *p < *0.01; *** = *p < *0.001 all relative to control values.

Since PLS-DA models are descriptive and not easily compared to other clinical statistics, individual metabolite information was collated. For each of the predictive models metabolites with high variable importance scores had their integral areas summed for the buckets of relevance (Table II, Figure 4). For each metabolite a one-way ANOVA with Dunnet's post hoc test was used to determine if the metabolite abundance was different from control values. Lactic acid and citric acid were increased in all ALS groups with respect to control, while glucose was increased in all except the greater than five-year duration model. Ethanol was significantly increased in the ≥ 6 months and ≥ 12 months ALS patient groups but not increased significantly in the ≥ 18 month and duration ≥ 5 years model.

**Table II.** Metabolite changes relative to control for metabolites of interest in the models.

		Ctrl		ALS ≥ 06		ALS ≥ 12		ALS ≥ 18		> 5 years	
Metabolite	Bucket region	Mean	SD	Mean	SD	Mean	SD	Mean	SD	Mean	SD
Lactic acid	[1.32...1.34] + [4.10...4.14]	1.00	0.14	1.22	0.16	1.22	0.16	1.21	0.18	1.23	0.19
Citric acid	[2.64...2.66]	1.00	0.17	1.39	0.23	1.39	0.26	1.36	0.29	1.31	0.26
Glucose	[3.24...3.28] + [3.38...3.44] + [3.46...3.52] + [3.54...3.56] + [3.70...3.80] + [3.82...3.86] + [3.88...3.92]	1.00	0.08	1.17	0.18	1.19	0.19	1.20	0.21	1.18	0.15
Ethanol	[1.18...1.20] + [3.66...3.68]	1.00	0.09	1.31	0.38	1.30	0.36	1.18	0.10	1.17	0.14

## Discussion

This study demonstrated that proton NMR spectroscopy, together with PLS-DA, has the potential to distinguish ALS patient and healthy control CSF metabolite profiles across a longitudinal cohort. The statistical models generated showed significant separations when comparing the more advanced of the longitudinal patient samples. Metabolites identified as discriminating ALS patients from controls (including glucose, lactate, citric acid and ethanol) were common to all models, supporting the view that they reflect consistently and progressively deranged metabolic pathway perturbations. Validation of the descriptive PLS-DA models using ANOVA generally showed agreement between the methods when assigning significance to the metabolite changes. Differences between the methods can be attributed to the nature of the PLS-DA models which calculate contribution to a model differently to the way the ANOVA calculates significance. The PLS-DA models show the contribution of the metabolite in the context of the complete set present in the biofluid while the ANOVA works in isolation.

Increases in metabolites such as glycolytic and citric acid cycle intermediates, together with creatine, ascorbate, acetone, glutamate and β-hydroxybutyrate have been reported in other cross-sectional studies in ALS (3,4,8), together with reductions in the levels of histidine, threonine, and creatinine (4,8,17). Serum glutamate was reported to be linked to disease duration (4), which fits with a long-established concept of excitotoxicity in ALS pathogenesis (18). The CSF increases in glucose, lactate and citric acid identified in the current study suggest significant alterations in energy metabolism. Evidence for disrupted mitochondrial function in ALS models has been frequently reported (19). A ‘hypermetabolic state’ is a recognized feature of ALS (20,21), and has been related to a broader concept of defective cellular energetic pathways (22). Such dysfunction may lead to increased energy requirements in the muscle and brain of ALS patients (23,24). This is predicted to result in increased production of citric acid, increased demand and consumption of carbohydrates such as glucose and increased production of glycolytic products such as lactate (8). A previously published study of the CSF metabolome in a larger number of ALS patients noted increases in pyruvate, also involved in energy metabolism, but not glucose or lactate as we report (3).

The detection of ethanol as a discriminating metabolite in the models produced from CSF of subjects affected with ALS was unexpected and has not previously been described. None of the participants had consumed alcoholic beverages on the day of study. Although it is possible that there was systematically greater use of recreational alcohol among our ALS patient cohort compared to controls, this has not been reported in other epidemiological studies of ALS (and reduced alcohol consumption has been identified as a risk factor (25)). We also note that a large proportion of the control samples came from cohabiting spouses of patients. To be certain that this ethanol was not a contaminant, a number of control experiments were performed (detailed in Supplementary information file to be found online at http://informahealthcare.com/doi/abs/10.3109/21678421.2015.1053490). In summary, no solvents, buffers or tubes were found to have ethanol contamination and any significant possibility of contamination from swabs was also eliminated.

We assume, therefore, that the observed peak reflects endogenous CSF ethanol, which is a well-established concept (26,27). Ethanol has been detected in the blood of people abstaining from alcohol consumption (28), and has been linked to metabolic disturbances, specifically involving mitochondria (29). The identification of endogenous ethanol might have particular relevance to ALS, where mitochondrial dysfunction is an area of intense research (19). Interestingly, endogenous CSF ethanol was also detected in the CSF of patients with cervical myelopathy, an occasional mimic disorder of ALS (30). The authors of that study drew a potentially unifying conclusion that the appearance of CSF ethanol is a result of tissue injury. Despite these observations, it is duly noted that two other NMR studies of the CSF metabolome in ALS, while detecting ethanol in one (3), did not note a significant difference between groups in either (3,17). In a ‘comprehensive’ CSF metabolome study in seven essentially healthy individuals being screened for meningitis, ethanol was not a reported metabolite, suggesting it is not routinely present (31).

Despite the positive findings reported here, it is important to note that our study does not replicate the previously published study in ALS (3). Further validation will be needed in other cohorts. It is also possible that the relatively low number of healthy control CSF samples limited the power of the modelling approach to detect subtle group-level metabolomic differences, while recruitment of patients at many different stages of disability prevented standardization of the ‘stage’ of disease at each time-point leading to greater noise in the data. At the same time, the progressive nature of ALS means that cohort studies tend to enrich for more slowly- progressive phenotypes, who are more likely to survive long enough to provide multiple sample time-points, in contrast to aggressive disease where only a baseline sample may have been provided. Consequently, the models with a greater gap between baseline and final sample contained progressively smaller numbers of patients, with a drop-off in separation from controls beyond the ≥ 12-months model. The subsequent improvement in separation between patients and controls in the ≥ 24-months model with the addition of the slowly-progressive PLS patients supports the view that statistical power was previously lacking. In contrast, the model with separation based on rate of disease progression did not reach significance. If our CSF metabolomic signature reflects a hypercatabolic state in ALS, then this might be evidence that it is inherent to the disease process, and not simply an effect of rapid accumulation of disability per se. Alteration in dietary intake associated with disease progression in ALS (e.g. gastrostomy supplementation) is another important potential confound not addressed in this study, but one that will need to be considered in future cohorts.

## Conclusions

The CSF metabolomic findings in a longitudinal ALS cohort support a model of profound changes in cellular energy metabolism during the symptomatic phase of the disease. Whether the apparent metabolic perturbation is a primary or secondary response to the disease, and whether it is specific to ALS (as, for example, might be seen in cancer- related cachexia), is not yet clear. Extension to the study of CSF in pre-symptomatic carriers of high-risk genetic mutations linked to the development of ALS is an important future initiative that will further define the diagnostic potential of the approach (32). Similarly, prospective studies comparing ALS patient cohorts with established disease mimics including undiagnosed cases of acquired neuromuscular weakness will be key extensions to the current study. More broadly, it is possible that neurochemical surrogates of deranged cellular metabolism in ALS, in particular our novel observation of endogenous CSF ethanol, might provide valuable pharmacodynamic biomarkers in future therapeutic trials.

## Supplementary materials available online

Supplementary Figure 1 and 2 to be found online at http://informahealthcare.com/doi/abs/10.3109/21678421.2015.1053490.
