# Supplementary figures and images for: The longitudinal cerebrospinal fluid metabolomic profile of amyotrophic lateral sclerosis
Source: Amyotroph Lateral Scler Frontotemporal Degener. 2015 Jun 29;16(7-8):456–63. doi: 10.3109/21678421.2015.1053490 (PMC4720042; doi:10.3109/21678421.2015.1053490)

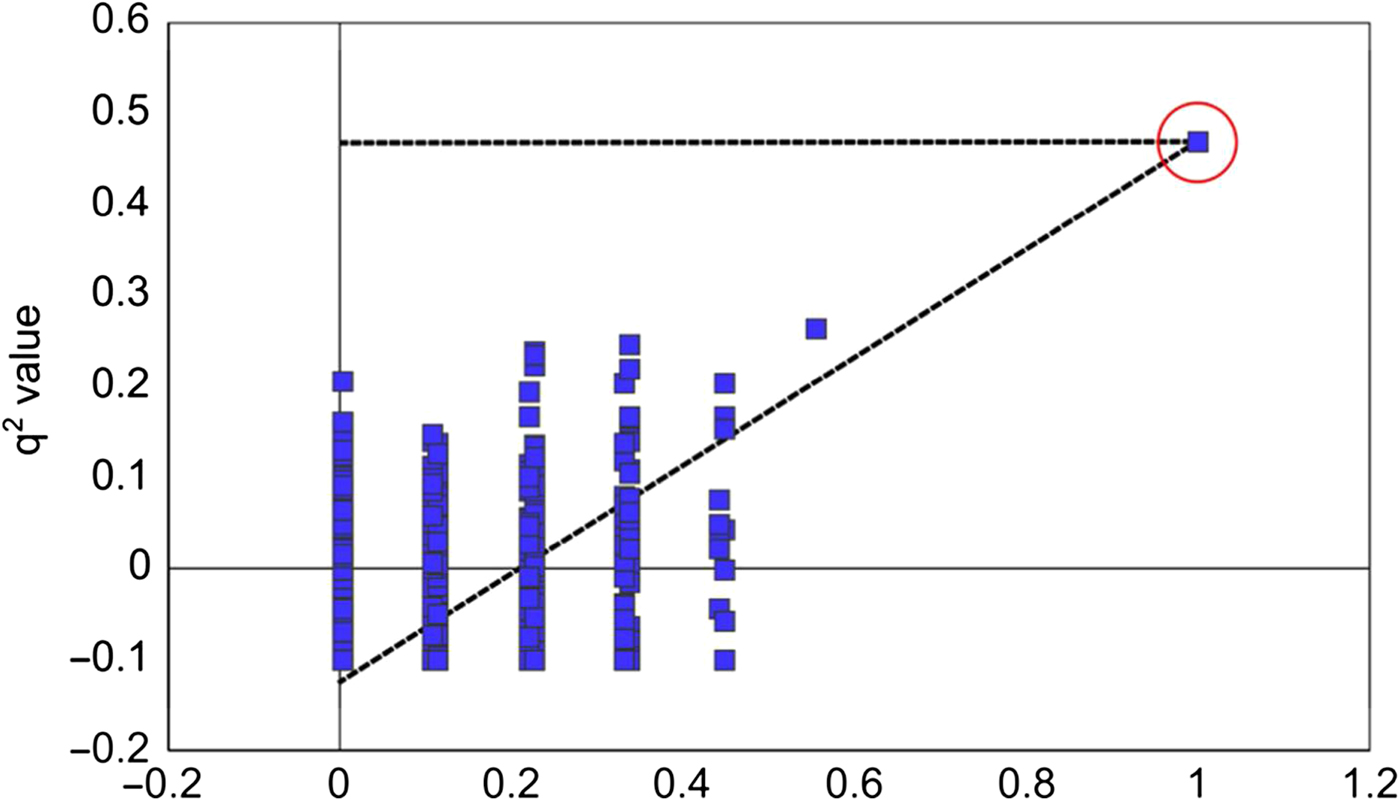

Supplement: Supplementary file 2 [file iafd_a_1053490_sm8059.tif]
