# Supplementary material for: The longitudinal cerebrospinal fluid metabolomic profile of amyotrophic lateral sclerosis
Source: Amyotroph Lateral Scler Frontotemporal Degener. 2015 Jun 29;16(7-8):456–63. doi: 10.3109/21678421.2015.1053490 (PMC4720042; doi:10.3109/21678421.2015.1053490)
Supplement: Supplementary file 3 [file iafd_a_1053490_sm8058.docx]

**Ethanol control experiments**

Owing to the possibility of ethanol contamination from laboratory glassware and reagents, and in light of a confirmed methanol contaminant, a number of control experiments were performed to eliminate the possibility that ethanol was a contaminant.

All solvents and buffers, as well as D_2_O rinsate from all glass and plasticware used, were analysed by NMR to confirm the absence of ethanol. The only liquids used in preparation of the samples were sterilization solutions. In our clinic, two different povidone-iodine preparations were in simultaneous use during the time of sample collections (Videne® antiseptic solution and Videne® alcoholic tincture). When analysed by NMR, the antiseptic solution did not contain ethanol but the alcoholic tincture did contain ethanol. To ascertain whether the alcoholic tincture was a source of contamination or not, clean dry skin was swabbed with the scrub and allowed to air dry, as happens during a lumbar puncture. Once dry, an area of skin was washed with 600 µl clean D_2_O into a tube and analysed by NMR. To magnify any effect that may have been present, the swabbed area was around 1000 mm², which is approximately 2500 times larger than the cross-sectional area of the 22-guage needle used for the lumbar punctures (0.4 mm²).

In this much exaggerated condition, there was a detectable ethanol peak, but it was still smaller than the peaks detected in CSF samples and smaller than one ten thousandth of the peak from the tincture before swabbing. This, combined with the fact that patients’ backs were randomly sterilized with either the alcoholic tincture or antiseptic solution, demonstrated that the ethanol peaks we observed in the CSF samples were genuine metabolites and not an artefact of preparation.
